# Red and processed meat consumption and risk of ovarian cancer: a dose-response meta-analysis of prospective studies

**DOI:** 10.1038/bjc.2011.49

**Published:** 2011-02-22

**Authors:** A Wallin, N Orsini, A Wolk

**Affiliations:** 1 Division of Nutritional Epidemiology, Institute of Environmental Medicine, Karolinska Institute, Box 210, Stockholm 171 77, Sweden

**Keywords:** cohort studies, meat, meta-analysis, ovarian cancer, prospective studies, systematic review

## Abstract

**Background:**

During the last decade, the epidemiological evidence on consumption of meat and risk of ovarian cancer has accumulated.

**Methods:**

We assessed the relationship between red and processed meat consumption and risk of ovarian cancer with a dose-response meta-analysis. Relevant prospective cohort studies were identified by searching the PubMed and EMBASE databases through 21 January 2011, and by reviewing the reference lists of retrieved articles. Study-specific relative risk (RR) estimates were combined using a random-effects model.

**Results:**

Eight cohort studies were included in the meta-analysis. The summary RR for an intake increment of 100 g per week was 1.02 (95% confidence interval (CI), 0.99–1.04) for red meat and 1.05 (95% CI, 0.98–1.14) for processed meat. For an intake increment of four servings per week, the summary RR of ovarian cancer was 1.07 (95% CI, 0.97–1.19) for red meat (100 g per serving) and 1.07 (95% CI, 0.97–1.17) for processed meat (30 g per serving).

**Conclusion:**

Results from this dose-response meta-analysis suggest that red and processed meat consumption is not associated with risk of ovarian cancer. Although a lower consumption of red and processed meat may offer protection against other types of cancer, other interventions are needed to reduce the risk of ovarian cancer.

Although ovarian cancer is less common among women than cancer of the breast and uterus, it has a higher case-fatality rate (Parkin *et al*, 2005; Ferlay *et al*, 2010). The poor prognosis is largely due to late diagnosis. Although most women with localised ovarian cancer are cured, 70–75% of cases are diagnosed with advanced-stage disease, with a 5-year survival of about 20–30% (Parkin and Iscovich, 1997). Many of the factors most consistently associated with ovarian cancer risk are not easily modified. The best recognised protective factors include oral contraceptive use and parity, while infertility, early age at menarche, late age at menopause and talc use have been associated with an increased risk (Sueblinvong and Carney, 2009). Thus, identification of modifiable life-style factors, including diet, could provide an opportunity for primary prevention.

In a recent meta-analysis of seven case–control and four cohort studies (Kolahdooz *et al*, 2010), the evidence on meat consumption in relation to ovarian cancer risk was summarised. However, in this meta-analysis based on risk estimates for the highest *vs* the lowest category of consumption, the interpretation of the summary relative risk (RR) can be problematic. Results from the individual studies are largely dependent on the range of exposure, which can vary considerably between studies conducted in different populations. For example, while ⩽3 servings per week was the lowest category in one of the studies included in the red meat analysis (Tavani *et al*, 2000), ⩾2 servings per week was the highest category of consumption in another study (Bertone *et al*, 2002). Consequently, the summary RR based on categorical comparison becomes a vague instrument to illustrate the magnitude and implications of an association.

In most of the analyses by Kolahdooz *et al* (2010), there was statistically significant heterogeneity when risk estimates from all studies were pooled, suggesting that it is inappropriate to draw conclusions from these results. As revealed by the stratification by study type, much of the heterogeneity was attributable to the inclusion of studies with different designs. Compared with case–control studies, prospective cohort studies provide a higher level of evidence because of lower susceptibility to recall and selection bias, and the pooled estimates from these studies should be valued higher.

A dose-response meta-analysis limited to prospective cohort studies offers a solution to the problems presented, and would be a valuable complement to the previous meta-analysis. From a public health perspective, dose-response meta-analyses can provide more useful estimates better quantifying associations between specified amounts of food and disease risk. Therefore, to clarify a potential association between red and processed meat and ovarian cancer risk, we performed a dose-response meta-analysis limited to prospective cohort studies only.

## Materials and methods

### Search strategy

Eligible studies were identified by searching the PubMed and EMBASE databases through 21 January 2011. The keywords *ovarian cancer* or *ovary* and *cancer* were used in combination with *meat, red meat, processed meat, pork, beef* or *foods*. Further, the reference lists of retrieved articles and relevant review articles were examined for additional relevant studies. No language restrictions were imposed. This systematic review was planned, conducted and reported in adherence to standards of quality for reporting meta-analysis (MOOSE) (Stroup *et al*, 2000).

### Eligibility criteria

Studies were included in the meta-analysis if they met the following criteria: (1) prospective cohort design; (2) the exposure studied was red meat or processed meat; (3) the outcome of interest was incidence of or mortality from epithelial ovarian cancer; and (4) RR with corresponding 95% confidence intervals (CIs) (or data to calculate these) were presented.

### Data extraction

Data extracted from each study included the first author's last name, publication year, country where the study was performed, study period, number of cases and cohort size, measure and range of exposure, variables adjusted for in the analysis, and RRs with corresponding 95% CIs for each category of consumption of red meat and/or processed meat. When several risk estimates were presented for each type of meat, the ones adjusted for the greatest number of potential confounders were used. The study quality was assessed using the Newcastle–Ottawa Quality Assessment Scale for cohort studies, with which each study is judged based on the selection of the study groups, the comparability of the groups, and the ascertainment of exposure and outcome (Wells *et al*, 2011). Data extraction was conducted independently by two authors (Wallin and Orsini), with disagreements resolved by consensus.

### Statistical analysis

We used the method proposed by Greenland and Longnecker (1992) and Orsini *et al* (2006) to compute the trend from the correlated log RR estimates across categories of consumption. We investigated a potential non-linear relationship using restricted cubic splines, but found no evidence of non-linearity. As the included studies used different units to report consumption (i.e., grams or servings), we rescaled consumption into servings per week. We used 100 g as the approximate average serving size for red meat, and 30 g for processed meat. The median level of consumption for each category was assigned to each corresponding RR estimate. When the median consumption per category was not presented in the article, the midpoint between the upper and lower boundary was used. If the lowest category was open-ended, the lower boundary was assumed to be zero. If the upper boundary of the highest category was not provided, we assumed it to be of the same amplitude as the preceding category. In addition, we performed a sensitivity analysis assigning different doses to the top categories (1.2–1.8 times the lower boundary). Statistical heterogeneity between studies was evaluated by using the *Q* and *I*^2^ statistics (Higgins and Thompson, 2002). Publication bias was evaluated with the use of a funnel plot and with the Egger regression asymmetry test (Egger *et al*, 1997). *P*<0.05 was considered statistically significant. All statistical analyses were performed with Stata software, version 10 (Stata Corp., College Station, TX, USA).

## Results

### Literature search

**Figure 1:**
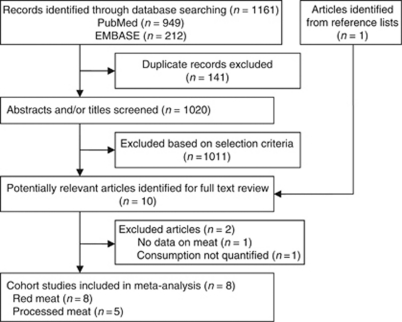
Flowchart of selection of studies for inclusion in meta-analysis.

A flowchart of the identification of relevant studies is shown in Figure 1. A total of 1161 articles were identified by searching the databases, 141 duplicated articles in the two databases and 1011 articles that did not meet the selection criteria were excluded. The remaining nine articles and one additional article (Cross *et al*, 2007) identified from reference lists were obtained for full-text review. Among these, one article was excluded because it did not present data on intake of meat in relation to risk of ovarian cancer (Chang *et al*, 2007), and one was excluded because meat consumption was not quantified (Knekt *et al*, 1994). The remaining eight prospective cohort studies were included in the meta-analysis (Kushi *et al*, 1999; Bertone *et al*, 2002; Larsson and Wolk, 2005; Kiani *et al*, 2006; Cross *et al*, 2007; Sakauchi *et al*, 2007; Schulz *et al*, 2007; Gilsing *et al*, 2011). Compared with the previous meta-analysis (Kolahdooz *et al*, 2010), we additionally included four cohort studies. The Adventist Health Study (Kiani *et al*, 2006) was included in the red meat analysis, as the reason for exclusion declared by Kolahdooz *et al* does not apply to a dose-response approach. We added also one American study that reported RR for unspecified meat (Kushi *et al*, 1999), assuming that the major part of this meat consumption is red meat. Moreover, one new study reporting on both red and processed meat consumption has been published (Gilsing *et al*, 2011). In addition, we included one study (Sakauchi *et al*, 2007) that reported risk estimates for red and processed meat consumption in relation to ovarian cancer mortality.

### Study characteristics

Characteristics of the included studies are presented in Table 1. The eight prospective cohort studies were published between 1999 and 2011 and involved a total of 754 836 participants and 2349 epithelial ovarian cancer cases. The outcome was incidence of ovarian cancer in seven studies (Kushi *et al*, 1999; Bertone *et al*, 2002; Larsson and Wolk, 2005; Kiani *et al*, 2006; Cross *et al*, 2007; Schulz *et al*, 2007; Gilsing *et al*, 2011), and mortality from ovarian cancer in one study (Sakauchi *et al*, 2007). Four studies were conducted in the United States (Kushi *et al*, 1999; Bertone *et al*, 2002; Kiani *et al*, 2006; Cross *et al*, 2007), 1 in 10 European countries (Schulz *et al*, 2007) and 1 each in Sweden (Larsson and Wolk, 2005), the Netherlands (Gilsing *et al*, 2011) and Japan (Sakauchi *et al*, 2007). Two studies were cohorts of only postmenopausal women (Kushi *et al*, 1999; Gilsing *et al*, 2011). The quality rating of the included studies ranged from five to eight stars on the scale of nine, with all but two studies meeting criteria for six stars (Bertone *et al*, 2002; Larsson and Wolk, 2005). All eight studies provided RR estimates adjusted for age, all but one study were further adjusted for parity (Cross *et al*, 2007), and all but two for body mass index (BMI) or waist-to-hip ratio (Bertone *et al*, 2002; Gilsing *et al*, 2011), and for total energy intake (Kiani *et al*, 2006; Sakauchi *et al*, 2007). Other covariates were less consistently used. All studies used self-administered food-frequency questionnaires (FFQ) to assess diet, and two studies updated the information with additional FFQs after baseline (Bertone *et al*, 2002; Larsson and Wolk, 2005). Two studies presented results for more than one relevant type of red meat (Bertone *et al*, 2002; Sakauchi *et al*, 2007). For those, we used the weighted average of the two estimates in the analysis of red meat consumption. The mean range of intake between the highest and the lowest category across studies was about 4.1 servings per week for red meat and about 5.5 servings per week for processed meat.

### Dose response of red and processed meat consumption

**Figure 2:**
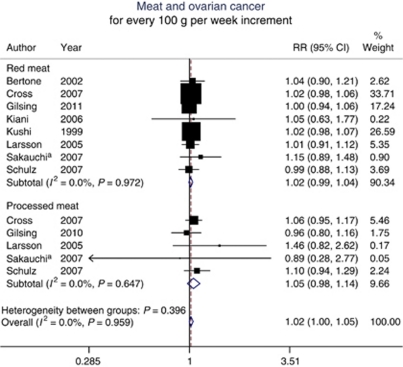
Relative risks of ovarian cancer associated with a 100 g per week increment in red or processed meat consumption. ^a^Outcome was mortality from ovarian cancer. Excluding this study did not appreciably change the results (RR for red meat, 1.02 (95% CI, 0.99–1.04); RR for processed meat, 1.06 (95% CI, 0.98–1.14); overall summary RR, 1.02 (95% CI, 1.00–1.05)).

**Figure 3:**
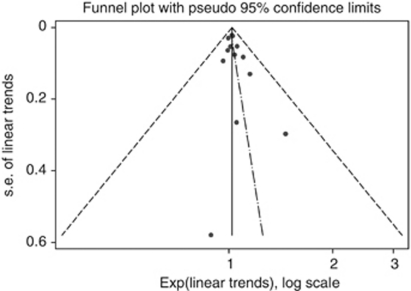
Funnel plot for evaluation of publication bias.

The summary RR for an intake increment of 100 g per week was 1.02 (95% CI, 0.99–1.04) for red meat and 1.05 (95% CI, 0.98–1.14) for processed meat. Combining the two types of meat resulted in an overall summary RR of 1.02 (95% CI, 1.00–1.05) (Figure 2). There was no heterogeneity between studies of red meat (*P*=0.97; *I*^2^=0.0%), between studies of processed meat (*P*=0.65; *I*^2^=0.0%), or in the overall summary estimate (*P*=0.96; *I*^2^=0.0%). For an intake increment of four servings per week, the summary RR of ovarian cancer was 1.07 (95% CI, 0.97–1.19) for red meat (100 g per serving) and 1.07 (95% CI, 0.97–1.17) for processed meat (30 g per serving). The Egger test showed no evidence of publication bias (*P*=0.20) (Figure 3).

In sensitivity analyses around the assignment of the dose of the top categories of consumption in studies that did not report median values, the summary estimates were not changed (RR=1.02 and RR=1.05 for every 100 g per week increment in consumption of red and processed meat, respectively). Excluding the largest study (Schulz *et al*, 2007), which accounted for 43% of the total number of participants and 25% of cases, did not appreciably change the results (RR=1.02 (95% CI, 0.99–1.05) and RR=1.04 (95% CI, 0.95–1.14)) for every 100 g per week increment in consumption of red and processed meat, respectively), neither did exclusion of the study that used ovarian cancer mortality as outcome (Sakauchi *et al*, 2007) (RR=1.02 (95% CI, 0.99–1.04) and RR=1.06 (95% CI, 0.98–1.14), respectively). In a further sensitivity analysis of red meat consumption, the summary RR was 1.01 (95% CI, 0.96–1.06) for studies that adjusted for oral contraceptive use (Larsson and Wolk, 2005; Sakauchi *et al*, 2007; Schulz *et al*, 2007; Gilsing *et al*, 2011), and 1.02 (95% CI, 0.99–1.06) for studies that did not (Kushi *et al*, 1999; Bertone *et al*, 2002; Kiani *et al*, 2006; Cross *et al*, 2007).

Our results are based on risk estimates for total epithelial ovarian cancer. Among the studies included in the meta-analysis, only three examined red and/or processed meat consumption in relation to histological subtype of ovarian cancer (Bertone *et al*, 2002; Larsson and Wolk, 2005; Schulz *et al*, 2007). There were no differences in observed associations between the subtypes within those studies.

## Discussion

The results from this meta-analysis of eight prospective studies suggest that red and processed meat consumption is not associated with risk of ovarian cancer. These results are partly in contrast to the conclusions from the previous meta-analysis, including both case–control studies and a smaller number of cohort studies, which reported a positive association with processed meat (Kolahdooz *et al*, 2010).

A strength of this study, in addition to the use of a dose-response approach, is that the assessment was based on data from prospective cohort studies only, which are less susceptible to recall and selection bias than retrospective case–control studies. Our findings also have limitations. First, a meta-analysis of observational studies cannot solve inherent problems with confounding in the included studies, which may introduce bias. Although most studies controlled for parity, BMI or waist-to-hip ratio, and total energy intake, other factors suspected to influence the risk of ovarian cancer were less consistently included in the multivariate models. Residual confounding by inadequately measured covariates could also be of concern. Second, our findings are likely to have been affected by misclassification of meat consumption because of imprecise measurement of diet in the included studies. In cohort studies, misclassification is generally non-differential, which most likely attenuates the association. Finally, because studies with null results or small sample sizes tend not to be published, publication bias, which may overestimate the summary RR, could be of concern. However, we found no evidence of publication bias in this meta-analysis.

Although the dose-response approach to meta-analysis offers advantages over the conventional methodology, it also requires a number of assumptions to be made. In terms of associations between absolute intake levels and disease risk, a meta-analysis can only be as good as the individual study instruments have been. All included studies used FFQs to assess meat consumption, which are generally more valuable to rank study participants according to their consumption than to measure exact levels. In addition, combining results from different studies requires comparing different instruments. Assumptions also had to be made regarding the median level of consumption for each category when this information was missing in the individual studies. However, sensitivity analysis around the assignment of the dose of open-ended top categories did not change the results. Finally, since some studies expressed consumption levels in servings rather than in grams, we had to make assumptions about average serving sizes.

Suggested mechanisms for a potential association between red and processed meat and ovarian cancer include high fat intake, which has been shown to be associated with elevated levels of circulating oestrogens (Hill *et al*, 1971; Aubertin-Leheudre *et al*, 2008). Although the role of oestrogen in ovarian cancer aetiology is not yet clear, the current evidence suggests that high levels may promote ovarian carcinogenesis (Lukanova and Kaaks, 2005). In a pooled analysis of 12 cohort studies, a weak positive association was observed for saturated fat intake, whereas other types of fat did not affect the risk of ovarian cancer (Genkinger *et al*, 2006). An earlier meta-analysis of primarily case–control studies found significant associations for total fat, saturated fat and animal fat (Huncharek and Kupelnick, 2001). Another possible mechanism is that preservation, cooking and/or processing methods can introduce mutagens and carcinogens to meat. These include N-nitroso compounds (NOC), heterocyclic amines and polycyclic aromatic hydrocarbons, many of which have been shown to induce tumours in several animal species and at several sites (Cross and Sinha, 2004). Haeme iron, which is more abundant in red meat than in white meat, has also been shown to stimulate endogenous NOC production (Cross *et al*, 2003). However, epidemiological studies have linked these compounds primarily to cancers of the gastrointestinal tract (De Stefani *et al*, 1998; Cross *et al*, 2010), whereas their potential role in ovarian cancer aetiology has not yet been investigated.

When investigating meat consumption, it has to be considered that any association with a diet high in meat may in part be attributable to a low intake of fruit and vegetables or other plant foods. Only two of the studies included in this meta-analysis controlled for fruit and vegetable consumption. Fruits and vegetables are high in antioxidants that have been shown to protect cells against oxidative damage and are hypothesised to reduce the risk of cancer (Thompson *et al*, 1999). In addition, plant foods contain phytoestrogens and fibre that may lower levels of circulating oestrogens (Adlercreutz, 1995; Aubertin-Leheudre *et al*, 2008). However, few prospective studies have shown significant associations between intake of fruit, vegetables or antioxidants and risk of ovarian cancer, although the overall evidence concerning vegetable consumption indicates a possible inverse association (Koushik *et al*, 2005, 2006). Another possibility is that a potential association between high meat consumption and risk of ovarian cancer may be mediated through obesity, as a result of high energy intake. Obesity influences endogenous hormones by increasing adrenal secretion of androgens, enhancing conversion of androgens to oestrogens and reducing plasma levels of sex hormone-binding globulin, which results in higher levels of biologically active oestrogen (Siiteri, 1987). In a meta-analysis of 28 studies, a moderate positive association between obesity and risk of ovarian cancer was suggested (Olsen *et al*, 2007). On the other hand, most of the studies included in our meta-analysis adjusted for BMI or waist-to-hip ratio and for total energy intake.

In conclusion, results from this dose-response meta-analysis suggest that red and processed meat consumption is not associated with risk of ovarian cancer. Considering the borderline significant results, a possible weak association cannot be excluded. However, ovarian cancer risk was only elevated by 2 and 5% for every 100 g increment in consumption of red and processed meat, respectively. Although a lower consumption of red and processed meat may offer protection against other types of cancer (Ferguson, 2010), other interventions are needed to reduce the risk of ovarian cancer.

## Figures and Tables

**Table 1:** Prospective cohort studies of red and processed meat consumption and ovarian cancer

**Study and location (follow-up)**	**Cases (cohort size)**	**Assessment of diet**	**Measure of exposure and range of exposure**	**RR (95% CI)**	***P* for trend**	**Covariates**	**Quality (0–9)** a
*Incidence*							
Kushi *et al*, 1999 United States (1986–1995)	139 (29 083 postmenopausal women)	Baseline FFQ	Meats: 9–12 servings per week *vs* <9 servings per week 13–17 servings per week *vs* <9 servings per week >17 servings per week *vs* <9 servings per week	1.18 (0.72–1.93)^b^ 0.68 (0.37–1.24)^b^ 1.60 (0.89–2.86)^b^	0.38	Age, energy intake, number of live births, age at menopause, family history of ovarian cancer, hysterectomy/unilateral oophorectomy, waist-to-hip ratio, physical activity, smoking, education	6
Bertone *et al*, 2002 United States (1980–1996)	301 (80 258)	FFQs, 1980, 1984, 1986, 1990	Hamburger: 1–3 servings per month *vs* <1 serving per month ⩾1 serving per week *vs* <1 serving per month Main dish of beef, pork, lamb: 1 serving per week *vs* <3 servings per month ⩾2 servings per week *vs* <3 servings per month	1.09 (0.83–1.44) 0.86 (0.63–1.17) 1.17 (0.91–1.51) 1.30 (0.93–1.82)	0.07 0.16	Age, parity, energy intake, age at menarche, menopausal status, postmenopausal hormone use, tubal ligation, smoking	5
Larsson and Wolk, 2005 Sweden (1987–2004)	288 (61 057)	FFQs, 1987, 1997	Red meat: 2 to <3 servings per week *vs* <2 servings per week 3 to <4 servings per week *vs* <2 servings per week ⩾4 servings per week *vs* <2 servings per week Sausage: ⩾0.5 to <2 servings per week *vs* <0.5 servings per week ⩾2 servings per week *vs* <0.5 servings per week	0.86 (0.62–1.21) 1.31 (0.94–1.82) 1.01 (0.70–1.46)^b^ 1.09 (0.87–1.36)^b^ 1.37 (0.83–2.24)^b^	0.27	Age, BMI, education, parity, use of oral contraceptives and postmenopausal hormones, energy intake, fruits, vegetables and dairy products	8
Kiani *et al*, 2006 United States, The Adventist Health Study (1976–1992)	71 (13 281)	Baseline FFQ	Beef: >0 to <1 servings per week *vs* never ⩾1 servings per week *vs* never	1.23 (0.67–2.25) 1.09 (0.50–2.38)	0.94	Age, parity, BMI, age at menopause, hormone replacement therapy	6
Cross *et al*, 2007 United States (1995–2003)	552 (119 312)	Baseline FFQ	Red meat: 21.4 g per 1000 kcal *vs* 9.8 g per 1000 kcal 31.4 g per 1000 kcal *vs* 9.8 g per 1000 kcal 42.9 g per 1000 kcal *vs* 9.8 g per 1000 kcal 62.7 g per 1000 kcal *vs* 9.8 g per 1000 kcal Processed meat: 4.4 g per 1000 kcal *vs* 1.6 g per 1000 kcal 7.6 g per 1000 kcal *vs* 1.6 g per 1000 kcal 12.3 g per 1000 kcal *vs* 1.6 g per 1000 kcal 22.6 g per 1000 kcal *vs* 1.6 g per 1000 kcal	1.20 (0.92–1.56) 0.97 (0.73–1.28) 1.09 (0.82–1.45) 1.19 (0.89–1.59) 1.14 (0.87–1.50) 1.21 (0.91–1.59) 1.13 (0.85–1.51) 1.23 (0.92–1.63)	0.33 0.30	Age, sex, education, marital status, BMI, family history of cancer, race, smoking, physical activity, energy intake, alcohol, fruit and vegetable consumption	6
Schulz *et al*, 2007 Europe (1992–2004)	581 (325 731)	Baseline FFQ	Red meat: 25 to <35 g per day *vs* <25 g per day 35 to <44 g per day *vs* <25 g per day 44 to <55 *vs* <25 g per day ⩾55 g per day *vs* <25 g per day Processed meat: 17 to <26 g per day *vs* <17 g per day 26 to <33 g per day *vs* <17 g per day 33 to <42 g per day *vs* <17 g per day ⩾42 g per day *vs* <17 g per day	1.22 (0.87–1.69) 1.13 (0.79–1.61) 1.13 (0.78–1.63) 1.04 (0.70–1.56) 0.98 (0.69–1.37) 1.10 (0.76–1.59) 1.09 (0.74–1.62) 1.25 (0.81–1.91)	0.89 0.23	BMI, parity, menopausal status, use of oral contraceptives, energy intake, education, smoking, unilateral ovariectomy, hormone replacement therapy	6
Gilsing *et al*, 2011 The Netherlands (1986–2002)	340 (62 573 postmenopausal women, including 2161 subcohort members for case–cohort analysis)	Baseline FFQ	Fresh red meat: 61.3 g per day *vs* 36.2 g per day 77.9 g per day *vs* 36.2 g per day 95.6 g per day *vs* 36.2 g per day 129.6 g per day *vs* 36.2 g per day Processed meat: 2.7 g per day *vs* 0 g per day 6.8 g per day *vs* 0 g per day 13.0 g per day *vs* 0 g per day 25.6 g per day *vs* 0 g per day	1.58 (1.08–2.30)^b^ 1.47 (1.00–2.16)^b^ 1.78 (1.23–2.58)^b^ 0.93 (0.61–1.42)^b^ 0.71 (0.49–1.03)^b^ 0.91 (0.64–1.29)^b^ 0.93 (0.65–1.31)^b^ 0.83 (0.59–1.20)^b^	0.85 0.74	Age, energy intake, parity, use of oral contraceptives	6
							
*Mortality*							
Sakauchi *et al*, 2007 Japan (1988–2003)	77 (63 541)	Baseline FFQ	Pork: 1–2 times per week *vs* ⩽1–2 times per month ⩾3–4 times per week *vs* ⩽1–2 times per month Beef: 1–2 times per month *vs* seldom ⩾1–2 times per week *vs* seldom Ham and sausage: 1–2 times per week *vs* ⩽1–2 times per month ⩾3–4 times per week *vs* ⩽1–2 times per month	1.26 (0.55–2.88)^b^ 1.59 (0.62–4.08)^b^ 1.06 (0.41–2.75)^b^ 1.24 (0.50–3.05)^b^ 0.73 (0.31–1.73)^b^ 0.91 (0.30–2.76)^b^	0.34 0.63 0.68	Age, menopausal status, number of pregnancies, sex hormone use (oral contraceptives and hormone replacement therapy), BMI, physical activity, education	6

Abbreviations: BMI=body mass index; CI=confidence interval; FFQ=food-frequency questionnaire; RR=relative risk.

a Evaluated according to the Newcastle–Ottawa Scale (Wells *et al*, 2011).

b Highest adjusted.
